# HAX-1 overexpression, splicing and cellular localization in tumors

**DOI:** 10.1186/1471-2407-10-76

**Published:** 2010-03-02

**Authors:** Alicja Trebinska, Alina Rembiszewska, Karolina Ciosek, Konrad Ptaszynski, Sebastian Rowinski, Jolanta Kupryjanczyk, Janusz A Siedlecki, Ewa A Grzybowska

**Affiliations:** 1 Cancer Center Institute, Roentgena 5, 02-781 Warsaw, Poland

## Abstract

**Background:**

HAX-1 has been described as a protein potentially involved in carcinogenesis and especially metastasis. Its involvement in regulation of apoptosis and cell migration along with some data indicating its overexpression in cancer cell lines and tumors suggests that HAX-1 may play a role in neoplastic transformation. Here we present the first systematic analysis of HAX-1 expression in several solid tumors.

**Methods:**

Using quantitative RT-PCR, we have determined the mRNA levels of *HAX1 *splice variant I in several solid tumors. We have also analyzed by semiquantitative and quantitative RT-PCR the expression of five *HAX-1 *splice variants in breast cancer samples and in normal tissue from the same individuals. Quantitative PCR was also employed to analyze the effect of estrogen on *HAX1 *expression in breast cancer cell line. Immunohistochemical analysis of HAX-1 was performed on normal and breast cancer samples.

**Results:**

The results reveal statistically important *HAX1 *up-regulation in breast cancer, lung cancer and melanoma, along with some minor variations in the splicing pattern. HAX-1 up-regulation in breast cancer samples was confirmed by immunohistochemical analysis, which also revealed an intriguing HAX-1 localization in the nuclei of the tumor cells, associated with strong ER status.

**Conclusion:**

HAX-1 elevated levels in cancer tissues point to its involvement in neoplastic transformation, especially in breast cancer. The connection between HAX-1 nuclear location and ER status in breast cancer samples remains to be clarified.

## Background

HAX-1 (HS1 associated protein X-1, encoded by the *HAX1 *gene) is an important target of study in the field of cancer research on account of its involvement in regulation of apoptosis and cell migration, key processes in carcinogenesis and metastasis.

The anti-apoptotic, cell-protecting properties of HAX-1 as well as its interactions with apoptosis-related proteins have been widely reported [1-3]. HAX-1 was demonstrated to interact with proteins involved in mitochondrial membrane permeabilization and elements of the mitochondrial mega-channel [4,5] as well as with proteins directly involved in initiation and execution of apoptosis [2,6,7] and with several viral proteins important for cell survival [1,8,9]. Recently, it was observed that HAX-1 is required for suppression of apoptosis in lymphocytes and neurons, by presenting Omi/HtrA2 to Parl processing [3]. Nevertheless, in spite of the large body of data indicating its role in apoptosis, molecular mechanisms of HAX-1-mediated cell protection still remain to be clarified.

The most explicit role of HAX-1 has been suggested by the studies on the pathogenesis of severe congenital neutropenia (Kostmann disease). This immuno-deficiency syndrome is characterized by the paucity of neutrophils in peripheral blood caused by a block in promyelocyte/myelocyte maturation, associated with their apoptosis. *HAX1 *mutations, leading to the inactivation of the protein, were found in Kostmann patients, indicating the involvement of HAX-1 in the functioning of the immunological system as well as in apoptosis [10].

HAX-1 multifunctionality manifests itself in a number of reported interactions with other proteins. One of these proteins is prohibitin. Prohibitin was initially identified as a repressor of estrogen-dependent transcriptional activity, but was subsequently shown to localize in the mitochondrial inner membrane and form a complex with VDAC, ANT2 and HAX-1, implying its role in apoptosis [5]. It has been reported that in the presence of ERα and estradiol, prohibitin translocates to the nucleus, providing a possible link between HAX-1 and estrogen-receptor signaling. Another possible connection to estrogen signaling is suggested by data from microarray analysis, which has classified *HAX1 *(as one of 172 out of 20,000 human genes) as estrogen-responsive [11,12]. Thus, further analysis of *HAX1 *responsiveness to estrogen seems to be worthwhile.

Besides its involvement in apoptosis, HAX-1 has been also implicated to function in regulation of cell migration [13,14]. The HAX-1 protein partner, HS1 [15], is highly homologous to cortactin, a cytoskeletal protein frequently overexpressed in cancer. Considering the similarity between HS1 and cortactin [16], it is not surprising that HAX-1 also binds the latter [13]. It has been suggested in several reports [17,18] that cortactin promotes tumor invasiveness and metastasis. While HS1 is expressed mostly in hematopoietic cells, cortactin is present in all other tissues [19,20]. It interacts with the Arp2/3 complex, promoting actin polymerization during actin network reconstruction in motile cells. HAX-1 was shown to form a complex with cortactin, the small GTP-ase Rac and regulatory protein Gα_13_. Accordingly, a model was proposed, in which Gα_13_, when bound to HAX-1, stimulates migration, while in the absence of HAX-1 it activates cell adhesion [13]. A more recent report [14] demonstrates the role of HAX-1 in regulation of carcinoma cell migration and invasion via clathrin-mediated endocytosis of intergin α_v_β_6_. The interaction of HAX-1 with the IL-1α precursor which has been shown to regulate human endothelial cell migration *in vitro *[21] provides additional support for a role in regulation of cell migration.

Since the inhibition of apoptosis and the induction of cell invasiveness are crucial for carcinogenesis, it is logical to expect that HAX-1 overexpression in neoplastic cells should contribute to tumor resistance to apoptosis as well as to the enhancement of metastatic potential.

HAX-1 overexpression has been observed in lesional psoriasis, a chronic inflammatory disease in which differentiation of keratinocytes is disturbed due to abnormal resistance to apoptosis [22]. In the same report, HAX-1 was shown to be overexpressed in melanoma cell lines. HAX-1 overexpression was also observed in oral squamous cell carcinoma (SCC) samples [14]. HAX-1 up-regulation in skin cancer samples and cell lines, its involvement in pathology of skin disease and the chromosomal location of the *HAX1 *gene within the epidermal differentiation complex (chromosome 1q21) suggests its role in maturation of the human epidermis. This conclusion points to its possible involvement in development of melanoma.

Except for the reports demonstrating HAX-1 overexpression in SSC samples and a few cancer cell lines, to date, the only available results concerning its expression in cancer consist of data from microarray and SAGE analyses. According to Oncomine, [23], a cancer microarray database, *HAX1 *is overexpressed in hepatoma, lung cancer, lymphoma, melanoma, leukemia and myeloma - in order of decreasing statistical significance. Downregulation of expression was observed for brain cancer, ovarian cancer and seminoma. In breast cancer, statistically significant overexpression correlated with carcinoma grade. In a gene expression profiling study utilizing SAGE, *HAX1 *overexpression was demonstrated to be specifically induced by hypoxia in renal cell carcinoma (RCC) cells [24]. Although these reports indicate *HAX1 *overexpression in cancer, their reliability is not as high as in the focused study, so they still need to be verified by more systematic analysis.

*HAX1 *was shown to be expressed quite ubiquitously in human tissues, with relatively higher expression in testis, liver and skeletal muscle [25]. It has also been shown to be alternatively spliced, producing at least five splice variants with the same open reading frame in human cells [25,26]. Expression analysis ascertained so far for variants I and II in normal tissues [25] shows prevalent expression of splice variant I. The role of the other variants in processes like apoptosis, cell signaling and migration, remains unclear. Analysis of the potential variations in *HAX1 *splicing patterns in tumors may shed some light on the role of splice variants in cancer pathology.

The involvement of HAX-1 in processes crucial to carcinogenesis as well as demonstration of its overexpression in several tumor cell lines provides strong arguments for a detailed analysis of its role in neoplastic transformation and metastasis. In this report, we present for the first time a focused analysis of *HAX1 *expression in several solid tumors, identifying three malignant neoplasms (breast cancer, lung cancer and melanoma) in which *HAX1 *is significantly up-regulated. Detailed analysis of expression of five splice variants of *HAX1 *in breast cancer revealed tumor-specific variations in the pattern of splicing. HAX-1 elevated expression in breast cancer was confirmed at the protein level, by immunohistochemistry, which also revealed its nuclear localization in ER-positive tumors. Expression results were validated against clinical data, including stage and grade of tumor, receptor and nodal status. Additionally, the influence of estrogen on *HAX1 *expression was estimated in a breast cancer estrogen-responsive cell line (MCF-7) and found not significant.

## Methods

### Breast cancer samples

Breast cancer samples were obtained from 15 breast cancer patients (median age 64 years, range 32-81 years) undergoing surgery at the Cancer Center - Institute of Oncology in Warsaw. Samples were diagnosed according to histopathological reports as primary invasive carcinomas (ductal, lobular, papillary and mixed). Paired samples of breast carcinoma and normal tissue adjacent to the carcinoma were collected from each patient. These tissues are referred to as 'normal' breast tissues, although they cannot be regarded as completely 'healthy normal' specimens. A detailed description of the breast carcinomas of the 15 patients is shown in Table 1. This study was approved by the local ethics committee and the patients gave an informed consent about the usage of tissues for research purposes.

**Table 1:** Tumor characteristics of the 15 patients' primary carcinomas

Characteristic	n	%
Size (cm)		
< 2	5	33.3
> 2	10	66.7

Nodal status		
Positive	9	60
Negative	6	40

Histology		
Ductal	7	46.7
Lobular	3	20
Papillary	4	26.7
Mixed	1	6.6

Stage		
I	1	6.7
IIA	8	53.3
IIB	4	26.7
IIIA	2	13.3

Grade		
1	3	20
2	9	60
3	2	13.3

ER status		
Positive	9	60
Negative	6	40

PR status		
Positive	8	53.3
Negative	7	46.7

HER2 status		
Positive	7	46.7
Negative	8	53.3

### Oncology qPCR arrays

The following oncology qPCR arrays were purchased from OriGene Technologies (Rockville, MD, USA) and used to asses human *HAX-1 *mRNA expression levels: TissueScan Oncology Survey Tissue qPCR Array I, TissueScan Breast Cancer Tissue qPCR Array II, TissueScan Lung Cancer Tissue qPCR Array III, TissueScan Melanoma Tissue qPCR Array. All the clinical and pathological information associated with samples in each of the panels can be found on the OriGene website http://www.origene.com/geneexpression.

### Tissue sampling

Breast cancer and adjacent normal tissue samples were selected by a pathologist from surgically removed tissues of breast cancer patients immediately after surgery. Samples were flash-frozen, pulverized in liquid nitrogen using Microdismembrator II (Braun Biotech, Aylesbury, UK) and stored at -80°C prior to RNA isolation.

### RNA isolation and cDNA synthesis

RNA from 30 - 90 mg of pulverized breast cancer tissue and normal controls from the same patient was isolated using NucleoSpin RNA II kit (Macherey - Nagel, Düren, Germany). RNA from 5×10^5 ^MCF-7 or Hela cells was isolated using PureLink Micro-to-Midi Total RNA Purification Kit (Invitrogen, Carlsbad, CA, USA). The amount and purity of RNA were measured spectrophotometrically on NanoDrop ND-1000 (NanoDrop Technologies, Wilmington, DE, USA). Overall RNA integrity and quality was assessed on a denaturing agarose gel. Genomic DNA was removed from RNA samples by digestion with recombinant DNase I, RNase-free (Roche, Mannheim, Germany) according to the manufacturer's instructions. First strand cDNA was synthesized from 50 ng (for semi-quantitative PCR on patient samples), 200 ng (for quantitative PCR on patient samples) or 1 μg (MCF-7 and Hela cells) of total RNA using 25 ng oligo(dT)_18 _primers (Fermentas, Burlington, Canada) and SuperScript II, RNase H - Reverse Transcriptase (Invitrogen) according to the manufacturer's instructions. Reverse transcription was repeated at least twice to minimize the effects of possible differences in reverse transcription efficiency amongst samples.

### Reverse transcription semi-quantitative PCR

Expression of *HAX-1 *splicing variants in breast cancer versus normal tissue was analyzed using reverse transcription semi-quantitative PCR. Splice variant nomenclature is presented as in Carlsson et al. [25]. Primers for PCR were designed to amplify *HAX1 *variant I, II and III specifically, primers for variants IV and V have been described previously [26]. Primers for *GAPDH *were described elsewhere [27]. Primer sequences are presented in Table 2. The amount of cDNA template corresponding to 2.5 ng (for *HAX1 *variant I and *GAPDH*), 5 ng (for *HAX1 *variant II) or 10 ng (for *HAX1 *variant III, IV and V) of total RNA was amplified using Taq DNA Polymerase (Invitrogen), 200 nM dNTP (Fermentas) and 100 nM of the appropriate primers. PCR conditions were: 95°C (5 minutes) followed by cycles of 95°C (30 seconds), 57°C (30 seconds), and 72°C (30 seconds). Cycle numbers for linear amplification of *HAX1 *were as follows: variant I - 35, *HAX1 *variant II - 35, *HAX1 *variant III - 40, *HAX1 *variant IV and V - 40, *GAPDH *- 30 cycles. The experiment was independently repeated thrice.

**Table 2:** Primers' sequences

mRNA	forward primer	reverse primer
*HAX1 *variant I	5'-GACCTCGGAGCCACAGAGAT-3'	5'-GGTGCTGAGGACTATGGAAC-3'
GenBank: NM_006118		

*HAX1 *variant II	5'-GGACCTCGGAGCTTCAG-3'	5'-TGACTCAGGACCTGGAAGTT-3' (semi-qPCR)
GenBank: NM_001018837		5'-CCATATCGCTGAAGATGCTA-3' (qPCR)

*HAX1 *variant III	5'-GACCTCGGAGGTGAGA-3'	5'-CCATATCGCTGAAGATGCTA-3'
GenBank: EU190983		

*HAX1 *variant IV	5'-AGGAATTTGGCTTCGGCTTC-3' [26]	5'-TGCAGAAAGGTGGCAGGTGTT-3' [26]
GenBank: EU190982 and *HAX1 *variant V VEGA:OTTHUMT00000087654		

*CTSD*	5'-GCTGTGAGGCCATTGTGGAC-3'	5'-GCGACACCTTGAGCGTGTAG-3'
GenBank: NM_001909		

*GAPDH*	5'-GGTCGGAGTCAACGGATTTG-3' [27]	5'-ATGAGCCCCAGCCTTCTCCAT-3' [27]
GenBank: NM_002046		

*ACTB*	5'-AGCCTCGCCTTTGCCGA-3' [28]	5'-GCGCGGCGATATCATCATC-3' [28]
GenBank: NM_001101		

Sequences of the primers used in reverse transcription semi-quantitative and quantitative PCR. GeneBank accession numbers are indicated for each transcript except for variant V where transcript ID from Vertebrate Genome Annotation (VEGA) database is shown.

### Reverse transcription quantitative PCR

Expression levels of *HAX1 *splicing variants in oncology qPCR arrays and breast cancer samples versus normal tissues as well as transcripts levels in cells treated with beta-estradiol were assessed using quantitative PCR on an ABI Prism 7000 Sequence Detection System (Applied Biosystems, Foster City, CA, USA) with human *ACTB *as a reference gene. Amplification mixtures (25 μl) contained 10 ng (samples obtained from patients) or 20 ng (MCF-7 and Hela cells) of cDNA template, 1×SYBR Green I Master Mix Buffer (Applied Biosystems) and 100 nM of the appropriate forward and reverse primer. Primers used for amplification of *HAX1 *variant I were the same as in semi-quantitative PCR. Primer sequences for *HAX1 *variant II and cathepsin D (*CTSD*) are shown in Table 2. Primers for the reference gene *ACTB *were described previously [28]. The cycling conditions for *HAX1 *splicing variants were as follows - precycling hold at 95°C for 10 minutes, 40 cycles: 95°C for 30 seconds and 60°C for 60 seconds; for *CTSD *- precycling hold at 95°C for 10 minutes, 40 cycles: 95°C for 30 seconds, 57°C for 30 seconds, 72°C for 30 seconds; for *ACTB *- precycling hold at 95°C for 10 minutes, 40 cycles: 95°C for 30 seconds, 55°C for 30 seconds, 72°C for 30 seconds. To assess specificity, amplification products were subjected to melting curve analysis. Raw data were analyzed using ABI Prism 7000 SDS Software Version 1.1 (Applied Biosystems). Relative expression levels were calculated using efficiency-corrected Ct model which takes into account amplification efficiencies of each primer pair [29].

### Estrogen activation

MCF-7 estrogen-dependent breast cancer cells and HeLa (human cervical carcinoma) cells were preconditioned for a week in D-MEM (Invitrogen) with charcoal-stripped FBS (Sigma-Aldrich, MO, USA) and subjected to the treatment with the indicated concentrations of beta-estradiol (Sigma-Aldrich) for 48 h [30]. cDNA obtained from the treated cells was analyzed by qPCR with primers specific for *HAX1*, variants I and II (as above), *CTSD *and *ACTB*.

### Immunohistochemical analysis

Immunohistochemical stainings were performed on paraffin-embedded material after heat-induced epitope retrieval (HIER). A mouse monoclonal anti-HAX-1 antibody was used at a concentration of 1:75 (BD Transduction Laboratories, USA). Antigens were retrieved by heating the sections in 0.01 M citrate buffer (pH 6.0) 6 × 5 min. in a microwave oven at 700 W. Non-specific tissue and endogenous peroxidase reactivity were blocked with 10% BSA and 3% H_2_O_2_, respectively. The sections were incubated with primary antibodies overnight at 4°C. The binding of the primary antibody was detected by a Vectastain Elite ABC kit (Vector Laboratories, Burlingame, CA, USA) and DAB was used as a chromogen. Slides were counterstained with Mayer's hematoxylin. Normal mouse IgG of the same subclasses and concentrations as the primary antibody served as negative controls.

### Statistical analysis

Statistical analysis was performed using Statistica 6.0 (StatSoft, Inc., OK, USA). Nonparametric Mann-Whitney test and Wilcoxon test for matched pairs' analysis were used to analyze mRNA expression data. Fisher's exact test was used to determine statistical significance of the IHC data. A P-value of < 0.05 was regarded as statistically significant.

## Results

### *HAX1 *expression level is elevated in breast and lung cancers and in melanoma

**Figure 1:**
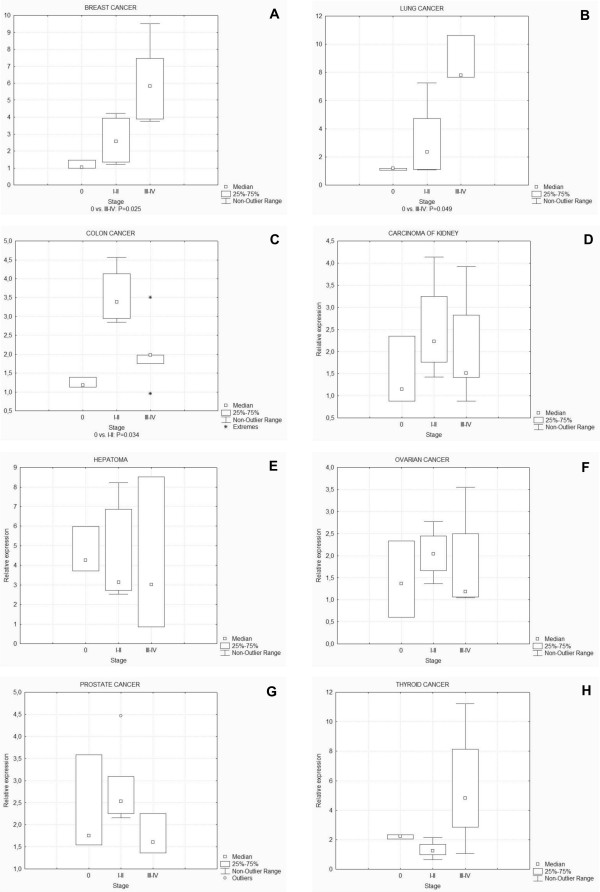
***HAX1 *(splice variant I) expression levels in eight different solid tumors**. *HAX1 *expression is significantly elevated in breast (A), lung (B) and colon (C) cancers, while in kidney (D), liver (E), ovary (F), prostate (G) and thyroid (H) cancers up-regulation was not detected. Quantitative data from cDNA panels were analyzed using the nonparametric Mann-Whitney test. Statistical significance was denoted under the box-whiskers plots for values of P < 0.05. In breast and lung cancers significant overexpression was observed for the advanced stages (III-IV), while in colon cancer for early stages (I-II).

In a preliminary screen, performed in order to asses *HAX1 *expression levels in different solid tumors, a survey panel containing cDNA from 96 tissue samples, covering 8 different cancers (breast, colon, kidney, liver, lung, ovarian, prostate, thyroid) was used (TissueScan Oncology Survey Tissue qPCR Array I). The samples represented disease stages from 0 to III or IV. Expression levels of all panel data were ascertained by quantitative PCR. Expression of splice variant I was assessed and considered as representative for overall *HAX1 *expression, on the basis of its prevalence in respect to other variants (as shown in this study in section "*HAX1 *splice variants expression in normal vs. tumor samples of breast cancer" and in Carlsson et al., 2008 [25]). Statistical significance for panel data was assessed using the nonparametric Mann-Whitney test. The highest levels of expression, significantly exceeding the level of normal control, were observed in breast and lung cancers (Fig 1A and 1B). In these two panels, up-regulation is significant in the most advanced stages of the disease (stages III-IV, P = 0.025 and 0.049 for breast and lung cancer, respectively). For the rest of the analyzed tumors, no significant overexpression or relation was observed, except for colon cancer, where significant up-regulation was detected for stages I-II (Fig 1C, D, E, F, G and 1H). Since our study was focused on finding a relationship between *HAX1 *expression levels and factors related to metastasis, such as stage, nodal status and grade of the disease, colon cancer was excluded from further analysis because of a non-significant p-level value for stages III-IV.

To further verify the results obtained from the survey panel, and to narrow the analysis, three other panels - breast cancer, lung cancer and melanoma - were analyzed, each containing cDNA from 48 tissue samples for each cancer type. Melanoma was included because of the potential role of HAX-1 in skin diseases and its reported overexpression in melanoma cell lines [22]. Panels contained samples from different stages of disease, including metastatic carcinomas (2, 7 and 40 cases for breast cancer, lung cancer and melanoma, respectively).

**Figure 2:**
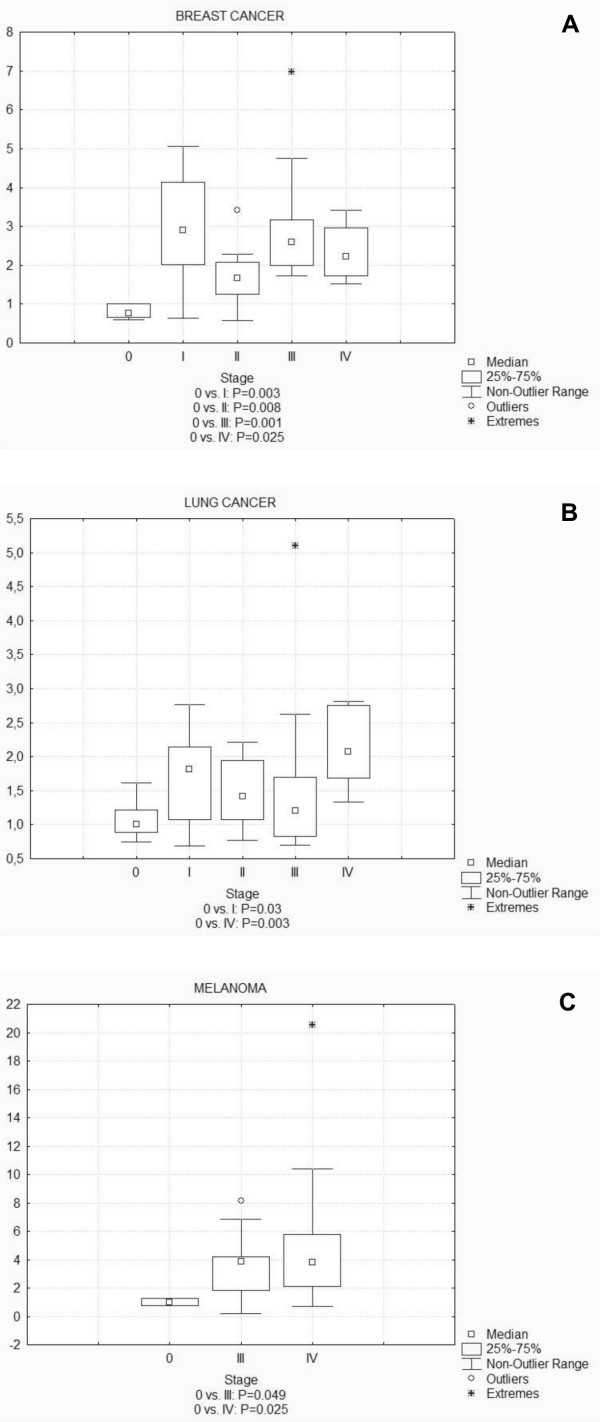
***HAX1 *(splice variant I) expression analysis in disease-focused panels representing breast cancer (A), lung cancer (B) and melanoma (C)**. Data were analyzed using non-parametric Mann-Whitney test and statistical significance for values of P < 0.05 was denoted under the plots. For breast cancer samples significant overexpression level was detected in all stages (I, II, III and IV). Analysis of the lung cancer samples show significant overexpression for stages I and IV. For melanoma, *HAX1 *expression in all analyzed stages was significantly up-regulated (only stages III and IV were analyzed).

Detailed analysis of the expression data obtained from these specific panels was performed using non-parametric Mann-Whitney test and confirmed *HAX1 *significant up-regulation in tumors, though not as high as previously detected in the survey panel (Fig 2). The most marked results were obtained for breast cancer samples, with significant overexpression (in regards to stage 0) for all subsequent stages of the disease (Fig 2A). In lung cancer samples, significant overexpression was obtained for stages I and IV, with higher expression levels and higher significance for stage IV (Fig 2B). The melanoma tissue panel was composed of normal and exclusively metastatic carcinoma samples from stages III and IV. Significant overexpression was obtained for both groups, with higher significance for stage IV (Fig 2C).

### *HAX1 *splice variants expression in normal vs. tumor samples of breast cancer

**Figure 3:**
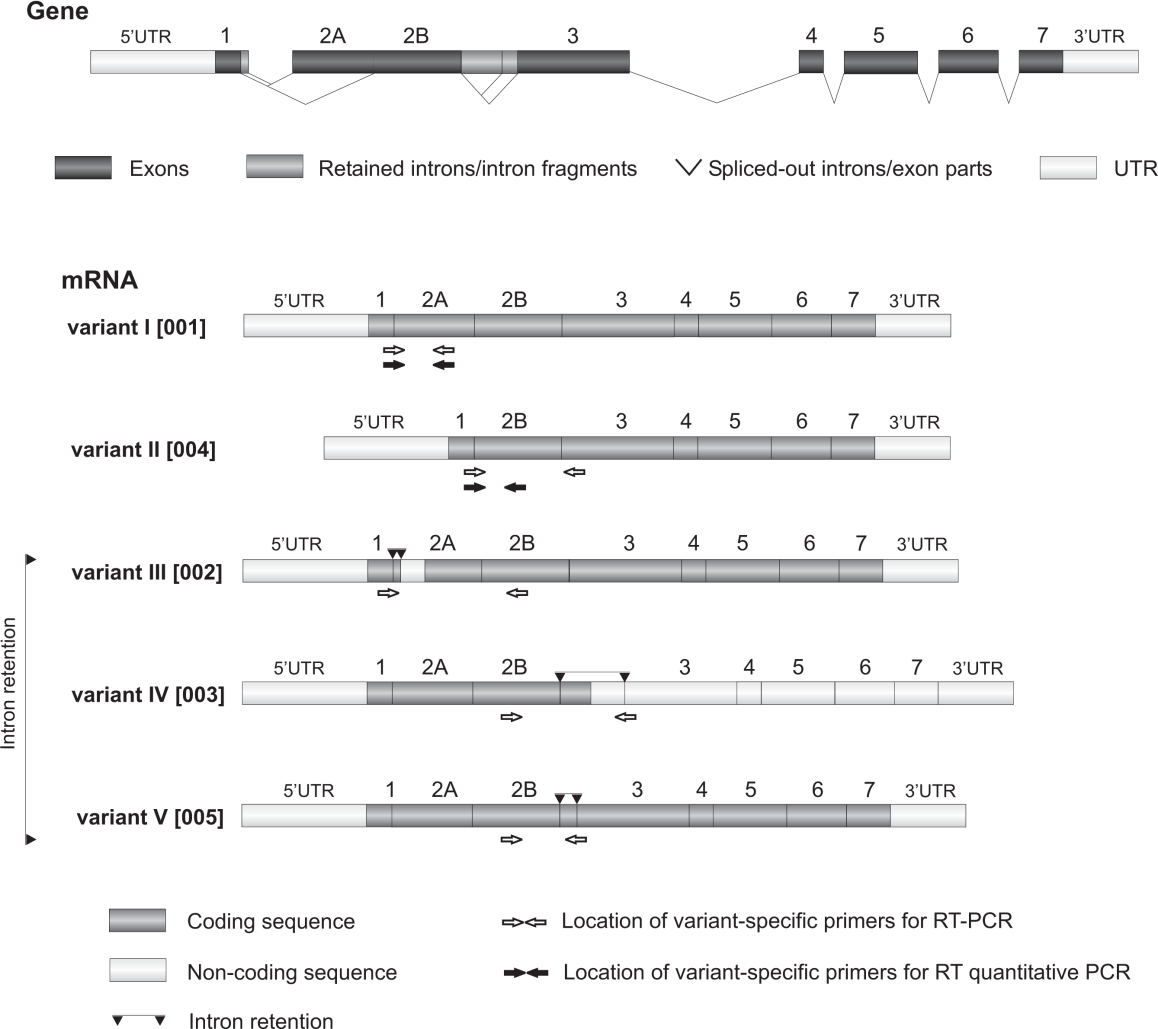
**Alternative splicing of the human *HAX1***. Only five splice variants, generating putative protein product maintained in the same reading frame are depicted. Variants are named as in Carlsson et al., 2008 [25], nomenclature from Lees et al., 2008 [26] in brackets. The location of the variant-specific primers is depicted by arrows.

Human *HAX1 *pre-mRNA is alternatively spliced to generate at least 8 splice variants [25,26]. Five of these variants remain in the same reading frame, while the rest contains a frameshift, generating a different protein product. In this analysis we ascertained the expression levels for the first five splice variants in the same reading frame (Fig 3).

**Figure 4:**
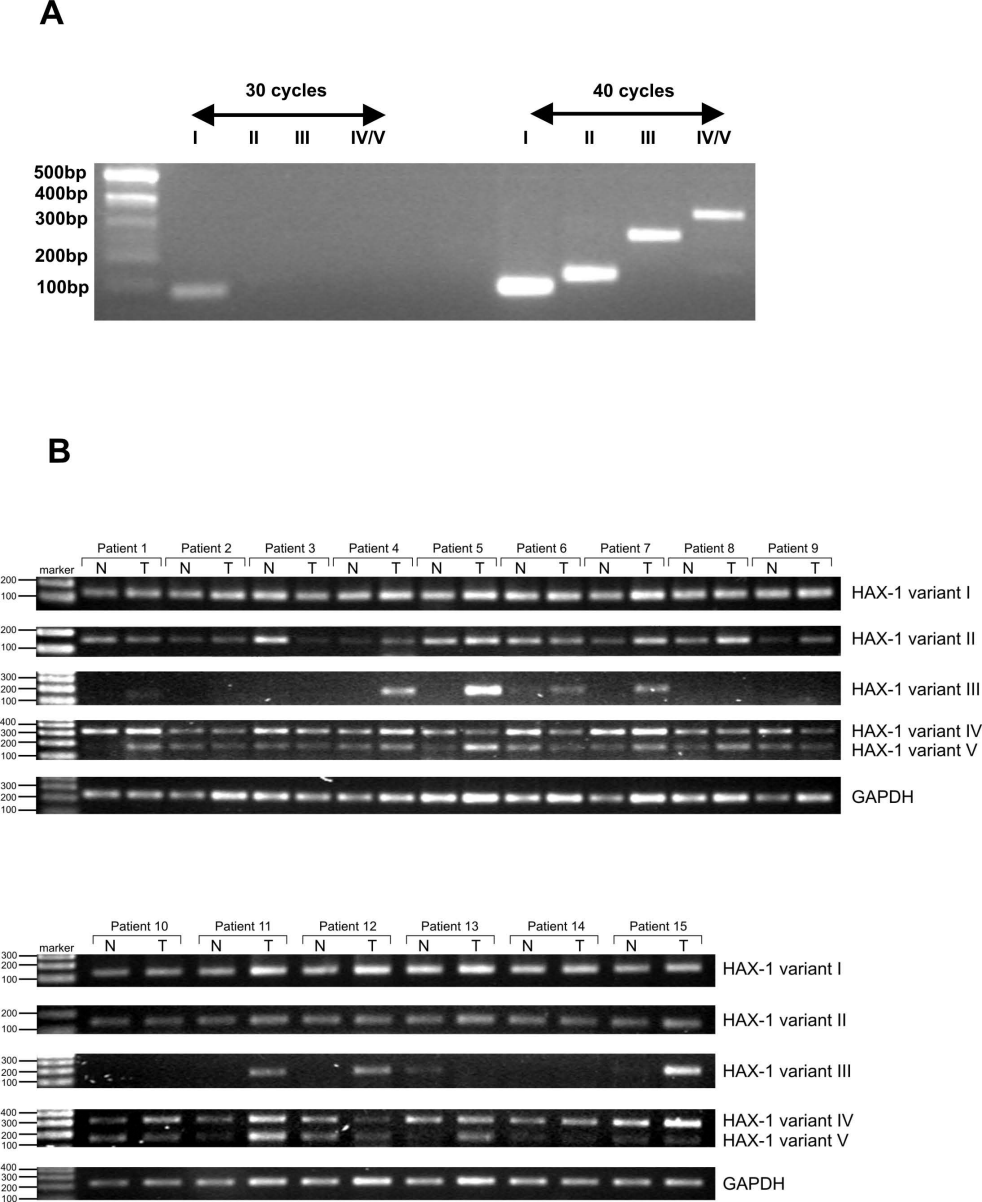
**Expression of the five *HAX1 *splice variants detected by semi-quantitative PCR**. A. Expression of the five *HAX1 *splice variants detected in the normal breast tissue reveals significant differences in expression levels, showing the prevalence of splice variant I. Normal breast tissue cDNA, splice variants I-V, 30 and 40 cycles. B. *HAX1 *splice variants expression in all 15 patients. The most marked difference was detected for variant III, the amplification of which was observed only in tumors in eight cases. In the remaining 7 cases no amplification was detected, except for patient 13, where a weak band was detected in the normal tissue sample, but not in the tumor sample. Higher amplification in tumors can be also observed in some patients for variant V (patients 1, 5, 7, 8, 11,13).

Since the previous study in which the expression of splice variants I and II was compared [25] revealed that variants' expression levels vary considerably (with the prevalence of splice variant I), to further demonstrate possible differences in expression the relative mRNA levels of the all five variants were assessed by standard RT-PCR. The results show that while variant I is quite abundant and easily detectable by 30 PCR cycles (Fig 4A), the expression of the other variants is quite weak (visible bands at 40 cycles, Fig 4A).

Samples of normal (adjacent to tumor) and tumor tissues from the same patient were analyzed for 15 breast cancer cases (Table 1). This type of material enables matched pair analysis, which eliminates differences in expression arising from the variability of genomic background. Expression of splice variants I, II, III, IV and V were analyzed by standard RT-PCR, with *GAPDH *as a reference (Fig 4B). The most marked difference in expression was detected in case of variant III. In 8 paired samples the expression of the splice variant III was detected only in tumors, while in the remaining 7 samples expression of variant III was detected neither in tumors nor in normal tissues, except for one case, in which the expression was detected in normal tissue and not in tumor. For the other variants the differences in expression were not detectable in the standard PCR experiment, apart from slight overexpression of variant V in six cases out of fifteen (Fig 4B).

**Figure 5:**
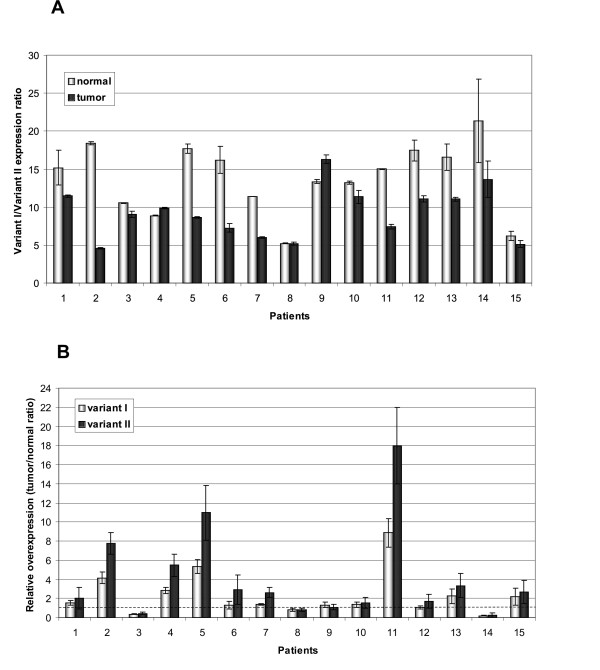
**Quantitative analysis of the expression of *HAX1 *variants I and II**. A. Expression of variant I is significantly higher than variant II, in both, normal and tumor tissues, but variant I/variant II expression ratio is higher in normal tissues (median 15) than in tumors (median 9), due to a relatively higher overexpression of variant II in tumors. B. Relative overexpression measured for variant I and variant II, calculated as a tumor/normal ratio. Overexpression, (expression higher than 1.5 fold) was found in 47% and 73% of cases for variants I and II, respectively. Dashed line indicates the level of expression in normal tissues. Order of patients according to the stage of the disease.

Expression of variants I and II was relatively high (although much higher for variant I), which made it possible to reliably and precisely assess its levels by quantitative PCR. For both variants up-regulation in tumors was statistically significant (P = 0.047 and P = 0.02 for variants I and II, respectively, nonparametric Wilcoxon test for matched pairs). The results also demonstrated, that although the high expression of the splice variant I in respect to the other variants (observed previously for the normal tissues, [25]) remains very high in tumors, the median value of the variant I/variant II expression ratio is considerably lower in tumor than in the matching normal tissues: 9 (6.63-11.23) and 15 (10.97-17.01), for tumor and normal tissues, respectively (Fig 5A). Accordingly, the relative tumor overexpression level in most cases (13 patients, 87%) is higher for variant II than for variant I (Fig 5B).

### Relationship between *HAX1 *expression and tumor characteristics

*HAX1 *expression levels were analyzed in respect to prognostic markers other than stage of the disease, in order to establish possible relationships. Factors like tumor size, grade of the disease, nodal status and histology were used to categorize data, followed by statistical analysis of the groups. For breast cancer, receptor status (ER, PR and HER2) was additionally included in the analysis. Data from the cDNA panel of breast cancer samples showed a significant relationship between *HAX1 *expression levels and tumor size (P = 0.004 and P = 0.0015 for T0 vs. T1 and T0 vs. T2-4, respectively) and grade (P = 0.003 and P = 0.0015 for G0 vs. G1-2 and G0 vs. G3, respectively), but for the other prognostic markers no significant relation was detected. For the other two panels, pathology reports concerning tumor size and grade were not sufficient to perform the analysis in the case of melanoma, and showed no relationship in the case of lung cancer.

Data from the matched pairs of normal and tumor tissue samples from the 15 patients were analyzed separately. The relationships between variants I and II expression levels and the established breast cancer prognostic factors were calculated and are summarized in Table 3. The most significant relationship was found, again, for tumor size (P = 0.005, same value for variants I and II). Relationships of weak significance (borderline) were found for nodal status (inverse relationship, variants I and II), progesterone-negative cancers (variants I and II) and HER2-negative cancers (variant II). Estrogen receptor status had no relation to *HAX1 *expression level, which confirms the results obtained from the panel data analysis.

**Table 3:** Relation of *HAX1 *expression to breast cancer prognostic factors

Characteristic	n	*HAX1 *(I) expression:	*HAX1 *(II) expression:
		median of relative over- expression (lower and upper quartile values)	median of relative over- expression (lower and upper quartile values)
Size (cm)			
< 2	5	0.82 (0.35-1.30)	0.82 (0.41-2.04)
> 2	10	2.2 (1.38-4.15)*	2.99 (1.70-7.5)*

Nodal status			
Positive	9	1.3 (1.54-4.15)	1.7 (0.82-2.90)
Negative	6	2.35 (0.82-2.18)	4.05 (2.04-7.75)*

Histology			
Ductal	7	1.3 (0.36-2.23)	2.04 (0.41-3.34)
Lobular	3	1.38 (1.33-2.83)	2.62 (1.54-5.50)
Papillary	4	3.77 (2.03-7.10)	6.81 (1.87-14.46)
Mixed	1	1.08	1.7

Grade			
1	3	1.3 (0.36-1.54)	2.04 (0.41-2.89)
2	9	1.88 (1.08-2.83)	2.62 (1.09-5.48)
3	2	3.17 (2.18-4.15)	5.2 (2.65-7.75)

ER status			
Positive	9	1.33 (1.08-1.54)	2.04 (1.55-2.89)
Negative	6	2.51 (1.88-4.15)	4.06 (1.09-7.75)

PR status			
Positive	8	1.32 (0.95-1.46)	1.9 (1.18-2.76)
Negative	7	2.23 (1.88-4.15)	3.34 (1.09-7.75)*

HER2 status			
Positive	7	1.30 (0.82-1.54)	1.7 (0.82-2.89)
Negative	8	2.51 (1.63-4.75)	4.06 (1.86-9.36)*

* denotes a P-value of < 0.005

Relationship between the expression of the *HAX1 *splice variants I and II and established prognostic factors in breast cancer. Median of relative overexpression was calculated for each group (lower and upper quartile values in parenthesis). Statistical significance was denoted in the groups were the number of cases was sufficient to perform the Wilcoxon matched-pairs test (size, nodal status, ER, PR, HER2 status) and a P-value of < 0.05 was observed.

### Estrogen does not regulate *HAX1 *mRNA levels

**Figure 6:**
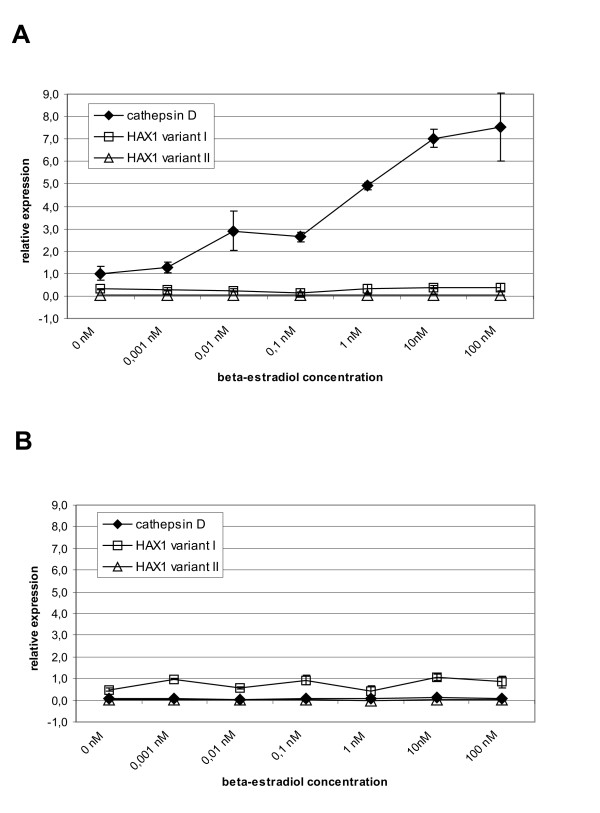
**Estrogen has no effect on *HAX1 *expression**. Preconditioned cells were treated with the increasing concentrations of beta-estradiol for 48 h. mRNA levels of *HAX1 *and estrogen-dependent cathepsin D mRNA were measured by quantitative PCR. A. Estrogen-dependent breast cancer cell line MCF-7. B. HeLa cell line.

The MCF-7 estrogen-dependent breast cancer cell line was subjected to treatment with the indicated concentrations of beta-estradiol (Fig 6A). Concurrently, the same experiment was performed in HeLa cells as an estrogen-independent reference (Fig 6B). The levels of *HAX1 *expression for the two splice variants (I, II) in estradiol-treated cells were established by quantitative PCR. The effect of estrogen was assessed in comparison to the known up-regulation of expression observed for cathepsin D mRNA [30]. No significant *HAX1 *up- or down-regulation was observed for any of the splice variants. This result is consistent with the lack of relation between *HAX1 *expression levels and the ER status, in the samples from cDNA panels and from patients (Table 2).

### HAX-1 overexpression in tumors and partial localization in cell nuclei was detected at the protein level

**Figure 7:**
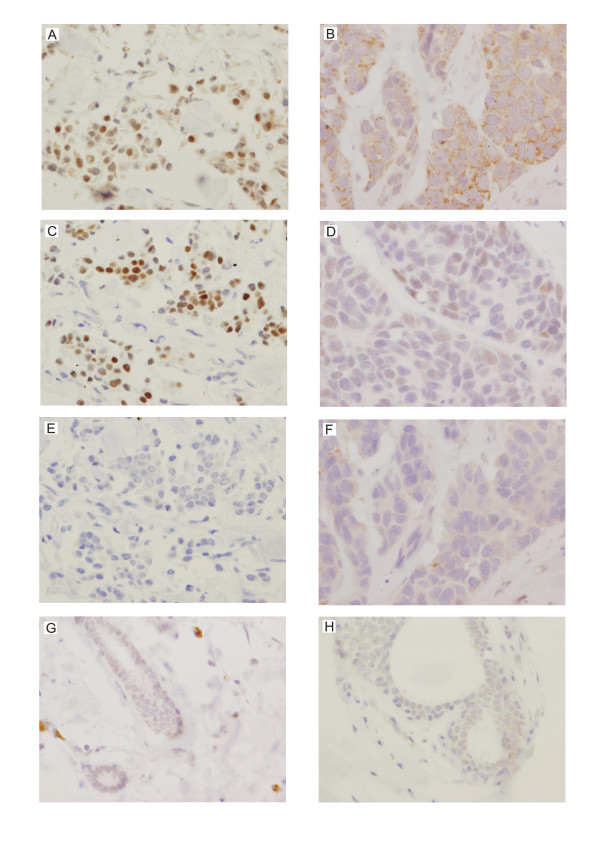
**Representative immunohistochemical staining of the analyzed breast cancer samples shows elevated levels of HAX-1 in tumor tissues**. Each column represents samples from one patient. HAX-1 staining was detected in the nuclei (A) and the cytoplasm (B) of tumor cells (magnification × 400). Nuclear HAX-1 localization was observed only in the strongly ER-positive samples (C), while in the samples with cytoplasmic HAX-1 staining ER expression was weak (D). Control samples incubated with mouse IgG of the same subclasses and concentrations as the primary antibody (E and F) were negative. Normal samples from the analyzed two patients (G and H) do not show visible HAX-1 staining.

*HAX1 *up-regulation detected at the mRNA level was confirmed at the protein level by immunohistochemical analysis. Paraffin sections of the paired normal and tumor tissues from all fifteen patients were analyzed. HAX-1 up-regulation was detected in most of the tumor samples (Table 4, Fig 7A and 7B). Intensive HAX-1 staining was observed not only in the cytoplasm (Table 4, Fig 7B), but also in the nuclei of the tumor cells (Table 4, Fig 7A). In the matching normal cells cytoplasmic staining was minimal (Table 4, Fig 7G and 7H) and nuclear staining was not detected (Table 4, Fig 7G). IgG controls of the analyzed tissue sections were negative (Fig 7E and 7F). It was observed, that strong nuclear HAX-1 staining was associated with strong ER staining (Table 4, Fig 7A and 7C), while cytoplasmic HAX-1 staining coincided with weak ER staining (Table 4, Fig 7B and 7D); this relation was calculated by the Fisher's exact test and found significant (P = 0.026).

**Table 4:** IHC analysis confirms HAX-1 overexpression in tumors

Patient	HAX-1				ER
	**Normal**		**Tumor**		**Tumor**

	**Cytoplasm**	**Nucleus**	**Cytoplasm**	**Nucleus**	**Nucleus**

1	0	-	2	-	+

2	0/1	-	1	-	-

3	1	-	1	-	-

4	0	-	0	-	-

5	0	-	1	-	-

6	0	-	1	-	-

7	1	-	0	+	+

8	0	-	2	+	+

9	0	-	1	-	-

10	2	-	2	-	+

11	1	-	1/2	-	+

12	0	-	2	+	+

13	0	-	2	-	-

14	0	-	2	+	+

15	0	-	3	-	-

Immunohistochemical analysis of the 15 matched pairs of normal/tumor samples shows in most cases significant overexpression of HAX-1 in tumors. Up-regulation was observed in cytoplasm as well as nuclei of the tumor cells. Staining intensity was estimated as follows: negative = 0; weak = 1; intermediate = 2; and strong = 3. Nuclear staining was categorized as negative or weak (0-1:"-") and intermediate-strong (2-3: "+"). Strong nuclear HAX-1 staining was associated with strong ER immunopositivity. Fisher's exact test indicates that nuclear staining of HAX-1 and ER is significantly related (P = 0.01).

HAX-1 nuclear localization was also observed by immunofluorescence in about 21% of cells in HAX-1-GFP-transfected MCF-7 breast cancer cell line, thus confirming IHC data, indicating that HAX-1 is present, at least to some extent, in the nuclei of breast cancer cells (Additional files 1 and 2).

## Discussion

HAX-1 protein has been reported to play a role in apoptosis and cell migration, its overexpression has been detected in cancer cell lines and - mostly by microarray analysis - in tumors, but hitherto, no systematic screen of its expression in cancer has been performed. This report represents the first preliminary analysis of *HAX1 *expression levels in solid tumors.

The initial survey, performed for a cDNA panel containing samples from eight different solid tumors revealed significant *HAX1 *overexpression for stages III-IV of breast and lung cancer. The samples derived from the other six cancers (colon, kidney, ovary, liver, prostate, thyroid) did not show significant changes in *HAX1 *expression, except for stages I-II of colon cancer.

An extended analysis of specific cDNA panels, containing a larger number of samples from different disease stages, confirmed the presence of statistically significant overexpression in breast and lung cancer and additionally, in melanoma. Detailed statistical analysis of these data has shown that for breast cancer and melanoma *HAX1 *is significantly overexpressed in subsequent stages, with higher significance for later stages. For breast cancer, a similar relation was observed for tumor size and histological grade, but no relation was detected for other prognostic markers such as nodal status and receptor status (ER, PR and HER2). The number of cases with metastatic disease for breast and lung cancer was not sufficient to calculate a relation between *HAX1 *overexpression and the presence of distant metastases. In the case of melanoma all tumor samples were derived from cancers with distant metastases, which also excluded the possibility of comparing the expression in primary and metastatic tumors. Detailed examination of the data from the lung cancer panel did not confirm high overexpression status detected previously in the survey panel, but nevertheless, a significant overexpression was observed for the samples from stages I and IV. Therefore, panel analysis provided evidence for the expected *HAX1 *overexpression in tumors and identified specific tumors in which such overexpression takes place. Our results indicate the relation of *HAX1 *expression to tumor size, stage and grade of the disease but more detailed analysis is needed to confirm if the observed relationships are in any way connected to a potential role for HAX-1 in metastasis. The lack of relationship between *HAX1 *expression and nodal status does not support such a hypothesis, but it still needs to be verified on a larger scale.

cDNA panel analysis produced results which are partially consistent with the microarray-generated data deposited in Oncomine (overexpression in lung cancer and melanoma, grade-specific overexpression in breast cancer), but there are also substantial differences (lack of significant overexpression in hepatoma).

To further analyze *HAX1 *up-regulation in breast cancer, matching pairs of tumor and normal adjacent tissues from 15 breast cancer patients were examined, confirming significant overexpression in tumor tissue. The differential samples under analysis represented several types of tumor (ductal, lobulary, papillary and mixed carcinomas) with different tumor size, nodal and receptor status. The group was too uniform in respect of grade and stage of the disease to confirm the relationship of these factors to *HAX1 *expression level. The analysis of other prognostic markers demonstrated a significant relation between *HAX1 *expression level and tumor size, which confirms the results obtained from the cDNA panel. No relation was detected for receptor status, again, confirming the results from the panel. The relationships found for nodal status, progesterone and HER2 status were of borderline significance and since they were not confirmed by the panel data, their reliability is doubtful. In general, data from matched-pair analysis are largely consistent with the data obtained from the cDNA breast cancer panel. The discrepancies are minor and can be explained by the small number of cases in the matched-pairs analysis.

Matched pairs of tumor and normal tissues were also used to determine the variations of *HAX1 *splicing pattern in breast cancer samples. Detailed quantitative analysis was possible only for splice variants I and II, because the expression of the other variants is barely detectable, and is therefore likely to produce ambiguous quantitative results. All five variants were however analyzed by standard RT-PCR. The results revealed that *HAX1 *splicing pattern in breast cancer is indeed slightly, but recurrently different than in normal tissues. Variant I expression remains high in normal and tumor tissues, compared to the expression of the other variants, but variant II levels are reproducibly higher in tumors (Fig 5), pointing to its specific up-regulation. The most marked difference in the splicing pattern was detected for splice variant III; its expression in normal samples could not be detected by standard RT-PCR, probably due to combined effects of its very low expression level and a relatively small amount of the obtained cDNA, but in more than a half of the samples a clear PCR product was detected in tumor samples (Fig 4). These results suggest that variant III expression could be tumor-specific. Up-regulation of the *HAX1 *alternative splice variants in cancer indicates their specific role, but cannot be explained until specific functions would be attributed to the different splice variants and their corresponding putative protein products.

HAX-1 overexpression in breast cancer samples was confirmed at the protein level by immunohistochemistry. Although the reaction is not standardized (as in the case of known markers), the expression was clearly up-regulated in almost all tumor tissue sections analyzed (Table 4). Overexpression was detected, as expected, in the cytoplasm, but also in the nuclei of tumor cells, which is inconsistent with previous findings, associating HAX-1 with cytoplasmic structures (ER and lammelipodia, [31]; ER and mitochondria, [1,15]; mitochondria, [7,9]. Suzuki et al., [15] observed HAX-1 at the nuclear envelope, but this location was never confirmed in subsequent reports. Location of Hax-1 in the nuclear matrix was previously detected in normal rat testis [32]. Nuclear localization of HAX-1 has also been observed in systemic sclerosis fibroblasts [33]. The latter represents so far the only report suggesting that this localization might be disease-dependent. Results presented here implicate, that the nuclear localization of HAX-1 might be also associated with some types of cancer.

HAX-1 overexpression detected in breast cancer, where estrogen receptor status is an obvious prognostic factor, combined with some literature data indicating possible induction of *HAX1 *by estrogen [11,12] provided reasons to ascertain the influence of the estrogen treatment on *HAX1 *expression. The results show clearly that, in spite of previous predictions, *HAX1 *mRNA expression level is estrogen-independent. This finding is in agreement with the lack of a relation between *HAX1 *expression levels and ER status in breast cancer samples. However, estrogen may regulate HAX-1 activity in some other, non-genomic way. The observed relation between nuclear localization of HAX-1 and estrogen receptor up-regulation suggests that estrogen may influence HAX-1 cellular localization. It has been observed [5] that HAX-1-interacting protein, prohibitin, is translocated to the nucleus upon estrogen treatment, and acts as a repressor of ER activity. It remains to be established if nuclear translocation of HAX-1 is also directly associated with estrogen activity, but the observed correlation points to this conclusion. There must be, however, some other factors affecting HAX-1 localization, since HAX-1-GFP-transfected MCF-7 breast cancer cells show nuclear localization of this fusion in about 21% of the analyzed cells (Additional Files 1 and 2), while estrogen treatment enhances nuclear localization only slightly, to about 26% (data not shown).

## Conclusions

This report provides evidence for HAX-1 up-regulation in several types of solid tumors, confirming previous estimations based on its anti-apoptotic activity, overexpression in cancer cell lines and scattered high-throughput data. These preliminary results call for a more detailed analysis to establish HAX-1 relation with tumorigenesis and a highly probable association with metastasis, along with clarification of the molecular mechanisms behind HAX-1 cellular functions.

## Abbreviations

3'UTR: 3' untranslated region; *ACTB*: beta-actin gene; ANT2: adenine nucleotide translocator 2; Arp2/3: actin-related protein 2/3; *CTSD*: cathepsin D gene; ERα: estrogen receptor alpha; *GAPDH*: glyceraldehyde-3-phosphate dehydrogenase gene; HS1: hematopoietic lineage cell specific protein 1; IHC: immunohistochemistry; Omi/HtrA2: Omi/high temperature requirement protein A2; Parl: presenilin associated rhomboid-like; qPCR: quantitative polymerase chain reaction; Rac: Ras-related small GTP-binding protein; RT-PCR: reverse transcription polymerase chain reaction; SAGE: serial analysis of gene expression; VDAC: voltage dependent anion channel.

## Competing interests

The authors declare that they have no competing interests.

## Authors' contributions

AT carried out the molecular genetic studies and participated in statistical analysis and drafting the manuscript, AR carried out the immunoassays, KC participated in molecular studies, KP carried out tissue sample selection and classification for immunohistochemistry, SR participated in immunoassays, sample collection and management, JK and JS participated in coordination of the study and helped to draft the manuscript, EG conceived of the study, participated in its design and coordination, carried out statistical analysis and drafted the manuscript. All authors read and approved the final manuscript.

## Pre-publication history

The pre-publication history for this paper can be accessed here:

http://www.biomedcentral.com/1471-2407/10/76/prepub

## Supplementary Material

Additional file 1 **HAX1 nuclear localization detected by immunofluorescence**. Methods and Results sections for detecting HAX-1-GFP fusion protein in transfected MCF-7 breast cancer cell line. Nuclear localization of the fusion protein was detected in about 21% of the cells. Click here for file

Additional file 2 **HAX1 nuclear localization detected by immunofluorescence **(Figure 1). Figure showing nuclear presence of the HAX-1-GFP fusion in MCF-7 breast cancer cell line. Click here for file

## References

[B1] Sharp TV Wang HW Koumi A Hollyman D Endo Y Ye H Du MQ Boshoff C K15 protein of Kaposi's sarcoma-associated herpesvirus is latently expressed and binds to HAX-1, a protein with antiapoptotic function Journal of virology 2002 76 2 802 816 10.1128/JVI.76.2.802-816.2002 11752170 PMC136811

[B2] Han Y Chen YS Liu Z Bodyak N Rigor D Bisping E Pu WT Kang PM Overexpression of HAX-1 protects cardiac myocytes from apoptosis through caspase-9 inhibition Circulation research 2006 99 4 415 423 10.1161/01.RES.0000237387.05259.a5 16857965

[B3] Chao JR Parganas E Boyd K Hong CY Opferman JT Ihle JN Hax1-mediated processing of HtrA2 by Parl allows survival of lymphocytes and neurons Nature 2008 452 7183 98 102 10.1038/nature06604 18288109

[B4] Matsuda G Nakajima K Kawaguchi Y Yamanashi Y Hirai K Epstein-Barr virus (EBV) nuclear antigen leader protein (EBNA-LP) forms complexes with a cellular anti-apoptosis protein Bcl-2 or its EBV counterpart BHRF1 through HS1-associated protein X-1 Microbiology and immunology 2003 47 1 91 99 12636258 10.1111/j.1348-0421.2003.tb02790.x

[B5] Kasashima K Ohta E Kagawa Y Endo H Mitochondrial functions and estrogen receptor-dependent nuclear translocation of pleiotropic human prohibitin 2 The Journal of biological chemistry 2006 281 47 36401 36410 10.1074/jbc.M605260200 17008324

[B6] Lee AY Lee Y Park YK Bae KH Cho S Lee do H Park BC Kang S Park SG HS 1-associated protein X-1 is cleaved by caspase-3 during apoptosis Molecules and cells 2008 25 1 86 90 18319618

[B7] Cilenti L Soundarapandian MM Kyriazis GA Stratico V Singh S Gupta S Bonventre JV Alnemri ES Zervos AS Regulation of HAX-1 anti-apoptotic protein by Omi/HtrA2 protease during cell death The Journal of biological chemistry 2004 279 48 50295 50301 10.1074/jbc.M406006200 15371414

[B8] Dufva M Olsson M Rymo L Epstein-Barr virus nuclear antigen 5 interacts with HAX-1, a possible component of the B-cell receptor signalling pathway The Journal of general virology 2001 82 Pt 7 1581 1587 11413368 10.1099/0022-1317-82-7-1581

[B9] Yedavalli VS Shih HM Chiang YP Lu CY Chang LY Chen MY Chuang CY Dayton AI Jeang KT Huang LM Human immunodeficiency virus type 1 Vpr interacts with antiapoptotic mitochondrial protein HAX-1 Journal of virology 2005 79 21 13735 13746 10.1128/JVI.79.21.13735-13746.2005 16227293 PMC1262574

[B10] Klein C Grudzien M Appaswamy G Germeshausen M Sandrock I Schaffer AA Rathinam C Boztug K Schwinzer B Rezaei N HAX1 deficiency causes autosomal recessive severe congenital neutropenia (Kostmann disease) Nature genetics 2007 39 1 86 92 10.1038/ng1940 17187068

[B11] Terasaka S Aita Y Inoue A Hayashi S Nishigaki M Aoyagi K Sasaki H Wada-Kiyama Y Sakuma Y Akaba S Using a customized DNA microarray for expression profiling of the estrogen-responsive genes to evaluate estrogen activity among natural estrogens and industrial chemicals Environmental health perspectives 2004 112 7 773 781 15159206 10.1289/ehp.6753 PMC1241992

[B12] Cicatiello L Scafoglio C Altucci L Cancemi M Natoli G Facchiano A Iazzetti G Calogero R Biglia N De Bortoli M A genomic view of estrogen actions in human breast cancer cells by expression profiling of the hormone-responsive transcriptome Journal of molecular endocrinology 2004 32 3 719 775 10.1677/jme.0.0320719 15171711

[B13] Radhika V Onesime D Ha JH Dhanasekaran N Galpha13 stimulates cell migration through cortactin-interacting protein Hax-1 The Journal of biological chemistry 2004 279 47 49406 49413 10.1074/jbc.M408836200 15339924

[B14] Ramsay AG Keppler MD Jazayeri M Thomas GJ Parsons M Violette S Weinreb P Hart IR Marshall JF HS1-associated protein X-1 regulates carcinoma cell migration and invasion via clathrin-mediated endocytosis of integrin alphavbeta6 Cancer research 2007 67 11 5275 5284 10.1158/0008-5472.CAN-07-0318 17545607

[B15] Suzuki Y Demoliere C Kitamura D Takeshita H Deuschle U Watanabe T HAX-1, a novel intracellular protein, localized on mitochondria, directly associates with HS1, a substrate of Src family tyrosine kinases J Immunol 1997 158 6 2736 2744 9058808

[B16] Uruno T Zhang P Liu J Hao JJ Zhan X Haematopoietic lineage cell-specific protein 1 (HS1) promotes actin-related protein (Arp) 2/3 complex-mediated actin polymerization The Biochemical journal 2003 371 Pt 2 485 493 10.1042/BJ20021791 12534372 PMC1223309

[B17] Li Y Tondravi M Liu J Smith E Haudenschild CC Kaczmarek M Zhan X Cortactin potentiates bone metastasis of breast cancer cells Cancer research 2001 61 18 6906 6911 11559568

[B18] Chuma M Sakamoto M Yasuda J Fujii G Nakanishi K Tsuchiya A Ohta T Asaka M Hirohashi S Overexpression of cortactin is involved in motility and metastasis of hepatocellular carcinoma Journal of hepatology 2004 41 4 629 636 10.1016/j.jhep.2004.06.018 15464244

[B19] Kitamura D Kaneko H Miyagoe Y Ariyasu T Watanabe T Isolation and characterization of a novel human gene expressed specifically in the cells of hematopoietic lineage Nucleic acids research 1989 17 22 9367 9379 2587259 PMC335138

[B20] Schuuring E van Damme H Schuuring-Scholtes E Verhoeven E Michalides R Geelen E de Boer C Brok H van Buuren V Kluin P Characterization of the EMS1 gene and its product, human Cortactin Cell adhesion and communication 1998 6 2-3 185 209 10.3109/15419069809004475 9823470

[B21] McMahon GA Garfinkel S Prudovsky I Hu X Maciag T Intracellular precursor interleukin (IL)-1alpha, but not mature IL-1alpha, is able to regulate human endothelial cell migration in vitro The Journal of biological chemistry 1997 272 45 28202 28205 10.1074/jbc.272.45.28202 9353269

[B22] Mirmohammadsadegh A Tartler U Michel G Baer A Walz M Wolf R Ruzicka T Hengge UR HAX-1, identified by differential display reverse transcription polymerase chain reaction, is overexpressed in lesional psoriasis The Journal of investigative dermatology 2003 120 6 1045 1051 10.1046/j.1523-1747.2003.12247.x 12787133

[B23] Rhodes DR Yu J Shanker K Deshpande N Varambally R Ghosh D Barrette T Pandey A Chinnaiyan AM ONCOMINE: a cancer microarray database and integrated data-mining platform Neoplasia (New York, NY) 2004 6 1 1 6 10.1016/s1476-5586(04)80047-2 PMC1635162 15068665

[B24] Jiang Y Zhang W Kondo K Klco JM St Martin TB Dufault MR Madden SL Kaelin WG Jr Nacht M Gene expression profiling in a renal cell carcinoma cell line: dissecting VHL and hypoxia-dependent pathways Mol Cancer Res 2003 1 6 453 462 12692265

[B25] Carlsson G van't Hooft I Melin M Entesarian M Laurencikas E Nennesmo I Trebinska A Grzybowska E Palmblad J Dahl N Central nervous system involvement in severe congenital neutropenia: neurological and neuropsychological abnormalities associated with specific HAX1 mutations Journal of internal medicine 2008 264 4 388 400 10.1111/j.1365-2796.2008.01982.x 18513342

[B26] Lees DM Hart IR Marshall JF Existence of multiple isoforms of HS1-associated protein X-1 in murine and human tissues Journal of molecular biology 2008 379 4 645 655 10.1016/j.jmb.2008.04.020 18472110

[B27] Brossart P Keilholz U Scheibenbogen C Mohler T Willhauck M Hunstein W Detection of residual tumor cells in patients with malignant melanoma responding to immunotherapy J Immunother Emphasis Tumor Immunol 1994 15 1 38 41 8110729 10.1097/00002371-199401000-00005

[B28] Riou P Saffroy R Comoy J Gross-Goupil M Thiery JP Emile JF Azoulay D Piatier-Tonneau D Lemoine A Debuire B Investigation in liver tissues and cell lines of the transcription of 13 genes mapping to the 16q24 region that are frequently deleted in hepatocellular carcinoma Clin Cancer Res 2002 8 10 3178 3186 12374686

[B29] Pfaffl MW A new mathematical model for relative quantification in real-time RT-PCR Nucleic acids research 2001 29 9 e45 10.1093/nar/29.9.e45 11328886 PMC55695

[B30] Westley BR May FE Oestrogen regulates cathepsin D mRNA levels in oestrogen responsive human breast cancer cells Nucleic acids research 1987 15 9 3773 3786 10.1093/nar/15.9.3773 3588310 PMC340781

[B31] Gallagher AR Cedzich A Gretz N Somlo S Witzgall R The polycystic kidney disease protein PKD2 interacts with Hax-1, a protein associated with the actin cytoskeleton Proceedings of the National Academy of Sciences of the United States of America 2000 97 8 4017 4022 10.1073/pnas.97.8.4017 10760273 PMC18134

[B32] Sarnowska E Grzybowska EA Sobczak K Konopinski R Wilczynska A Szwarc M Sarnowski TJ Krzyzosiak WJ Siedlecki JA Hairpin structure within the 3'UTR of DNA polymerase beta mRNA acts as a post-transcriptional regulatory element and interacts with Hax-1 Nucleic acids research 2007 35 16 5499 5510 10.1093/nar/gkm502 17704138 PMC2018635

[B33] Kawaguchi Y Nishimagi E Tochimoto A Kawamoto M Katsumata Y Soejima M Kanno T Kamatani N Hara M Intracellular IL-1alpha-binding proteins contribute to biological functions of endogenous IL-1alpha in systemic sclerosis fibroblasts Proceedings of the National Academy of Sciences of the United States of America 2006 103 39 14501 14506 10.1073/pnas.0603545103 16971486 PMC1599989

