# Supplementary material for: HAX-1 overexpression, splicing and cellular localization in tumors
Source: BMC Cancer. 2010 Mar 2;10:76. doi: 10.1186/1471-2407-10-76 (PMC2843675; doi:10.1186/1471-2407-10-76)
Supplement: Additional file 1 — HAX1 nuclear localization detected by immunofluorescence. Methods and Results sections for detecting HAX-1-GFP fusion protein in transfected MCF-7 breast cancer cell line. Nuclear localization of the fusion protein was detected in about 21% of the cells. [file 1471-2407-10-76-S1.PDF]

Additional file 1

HAX-1 nuclear localization detected by immunofluorescence

Methods

Immunofluorescence

MCF-7 cells were grown on Lab-Tek coverslips in DMEM (10% FBS) and transfected using Lipofectamine 2000 (Invitrogen). Plasmid used for transfection was generated from the full-length human *HAX1* cDNA variant I and cloned in frame in pEGFP-N1 plasmid (Clontech) to create C-terminal fusion with GFP. Cells were fixed in 4% formaldehyde, washed with PEM buffer (80 mM PIPES, 5 mM EGTA, 2mM $MgCl_2$), quenched with 0.1M ammonium chloride and permeabilized with 0.5% Triton X-100 in PEM. Cells were stained with DAPI, mounted and observed using standardized settings on Nikon Eclipse E-800 microscope with 100x oil immersion objective (Plan Fluor; Nikon).

Results

HAX-1 is present in the nuclei of transfected breast cancer cells

MCF-7 breast cancer cell line was transfected with HAX-1-GFP-encoding construct and the localization was observed 24 hours after transfection. 200 randomly selected cells were examined for nuclear localization of HAX-1-GFP-fusion protein, which was detected in about 21 % of the cells (Figure 1, Additional file 2). This result demonstrates nuclear HAX-1 localization by the independent method and thus supports IHC data showing that HAX-1 nuclear localization occurs in breast cancer cells.
