# Supplementary material for: HAX-1 overexpression, splicing and cellular localization in tumors
Source: BMC Cancer. 2010 Mar 2;10:76. doi: 10.1186/1471-2407-10-76 (PMC2843675; doi:10.1186/1471-2407-10-76)
Supplement: Additional file 2 — HAX1 nuclear localization detected by immunofluorescence (Figure 1). Figure showing nuclear presence of the HAX-1-GFP fusion in MCF-7 breast cancer cell line. [file 1471-2407-10-76-S2.PDF]

Additional file 2

HAX-1 nuclear localization detected by immunofluorescence (Figure 1)

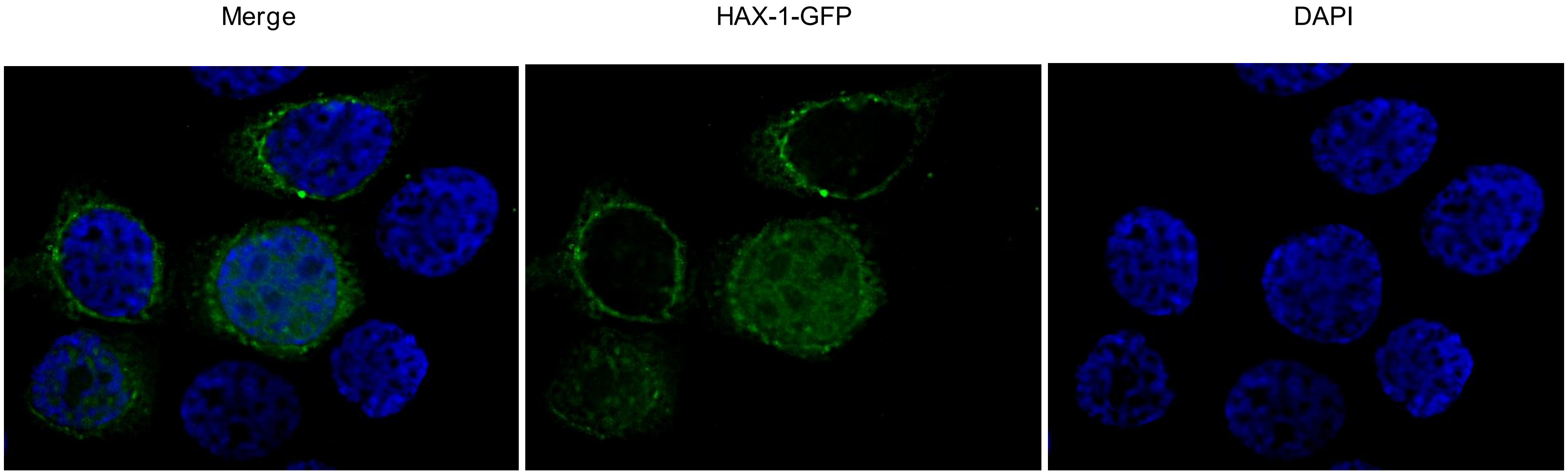

Figure 1. Immunofluorescence analysis reveals nuclear localization of HAX-1-GFP fusion protein in about 21% of MCF-7 cells. The remaining cells display cytoplasmic localization of the fusion protein. Images deconvolved with ImageJ image processing program [Abramoff MD, Magelhaes PJ, Ram SJ: **Image Processing with ImageJ**. *Biophotonics International* 2004, **11**(7):36-42].
